# Draft genome dataset of *Tapinoma indicum* (Forel) (Hymenoptera: Formicidae) in Penang Island, Malaysia

**DOI:** 10.1016/j.dib.2020.105903

**Published:** 2020-06-21

**Authors:** Li Yang Lim, Abdul Hafiz Ab Majid

**Affiliations:** Household & Structural Urban Entomology Laboratory, Vector Control Research Unit, School of Biological Sciences, Universiti Sains Malaysia, Penang, 11800 Minden, Malaysia

**Keywords:** *Tapinoma indicum*, Household ants, Genomic DNA, Whole-genome sequencing

## Abstract

*Tapinoma indicum* is a household pest that is widely distributed in Asian countries. It is known as nuisance pest that causes annoyance and disturbance by constructing nests and foraging in building for food and water. This article documents the draft genome dataset of *T. indicum* collected in Penang Island, Malaysia using the next-generation sequencing known as the Illumina platform. This article presents the pair-end 150 bp genome dataset and the quality of the sequencing result. This dataset provides the information for further understanding of T. *indicum* in the molecular aspect and the opportunity to develop a novel method for pest control and regulation. The dataset is available under Sequence Read Archive (SRA) databases with the accession number SRR10848807.

Specifications Table

**Table:** 

Subject	Biology
Specific subject area	Entomology, Genomics
Type of data	Genomic sequence
How data were acquired	Shotgun whole-genome DNA sequencing using the Illumina HiSeq platform
Data format	Raw sequencing data
Parameters for data collection	DNA extracted from five *Tapinoma indicum* workers’ head and thorax by removing the abdomen part.
Description of data collection	*Tapinoma indicum* was collected using the baiting method with peanut butter and honey. DNA was extracted using HiYield PlusTM Genomic DNA Mini Kit (Blood/tissue/cultured cells) (Real Biotech Corp., Taipei, Taiwan).
Data source location	Institution: University Sains Malaysia City/Town/Region: Penang Country: Malaysia GPS coordinate for the collected samples: N 5⁰21′7.20″, E 100⁰14′18.84″
Data accessibility	BioProject: PRJNA598521 (https://www.ncbi.nlm.nih.gov/bioproject/598521) BioSample: SAMN13707189 (https://www.ncbi.nlm.nih.gov/biosample/SAMN13707189) NCBI SRA: SRR10848807 (https://www.ncbi.nlm.nih.gov/sra/SRR10848807)

## Value of the data

• The first *Tapinoma indicum* sequenced draft genome data.
• *T. indicum* represents one of the major nuisance pests widely distribute in Asian countries.
• The *T. indicum* draft genome data could be used for microsatellite marker design.
• Further study could potentially develop a novel pest control and management approach based on genetic diversity of the pest (*T. indicum*).

## 1 Data description

The dataset described in this article is the whole-genome paired-end sequencing result of BioSample SAMN13707189 under the BioProject PRJNA598521. It is registered under the Sequence Read Archive (SRA) databases with the accession number SRR10848807. The data set comprised of two high throughput sequencing fastq files:

• TapinomaindicumR02read1.fastq;
• TapinomaindicumR02read2.fastq.

TapinomaindicumR02read1.fastq and TapinomaindicumR02read2.fastq contains half of the full sequence reads in a total of 16,363,685 raw reads with 150 bp each. TapinomaindicumR02read1.fastq makes up the 1st-150th base position while TapinomaindicumR02read2.fastq makes up the 151th-300th base position of each sequence.

**Fig. 1:**
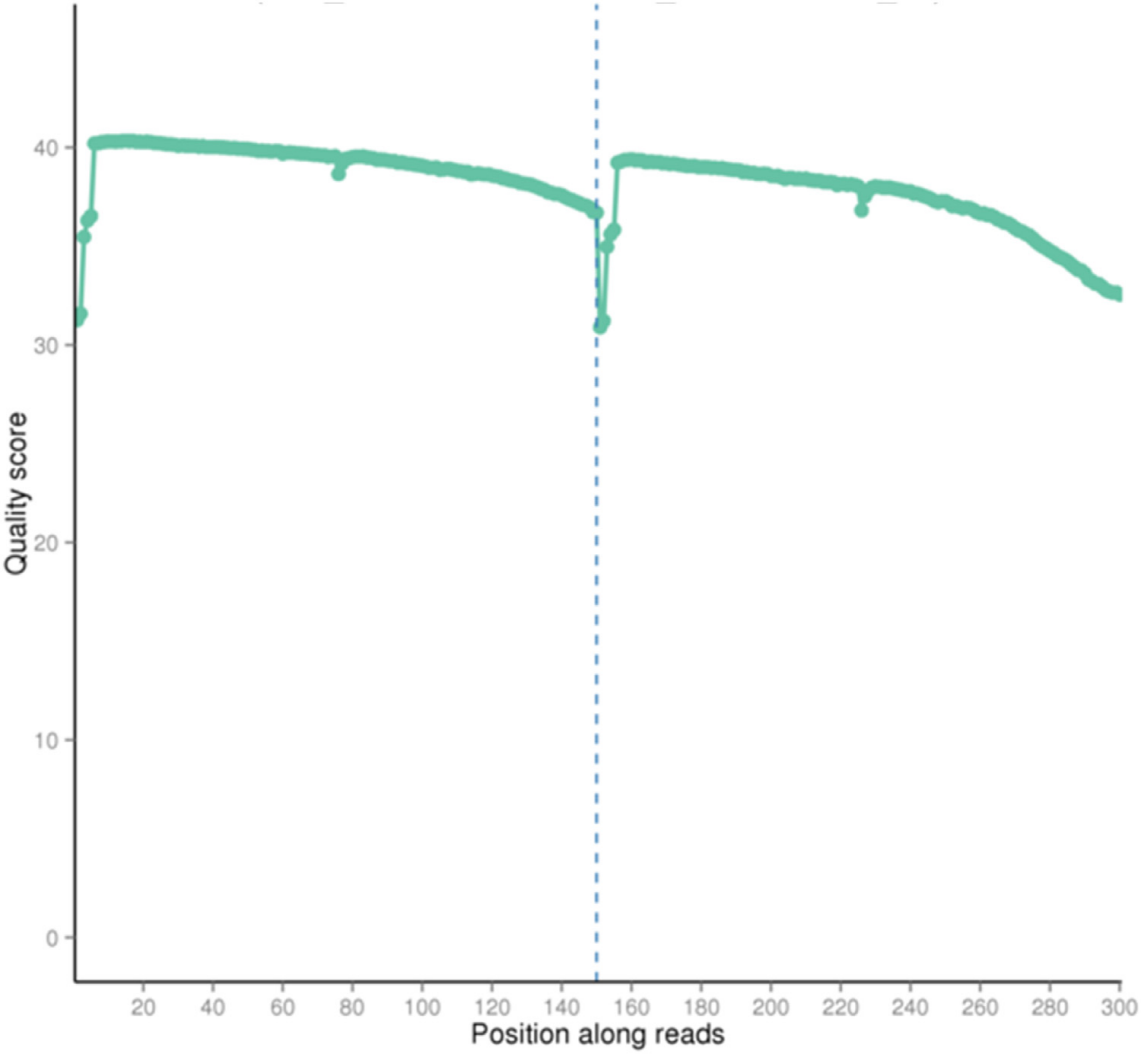
Quality score distribution along reads position.

**Fig. 2:**
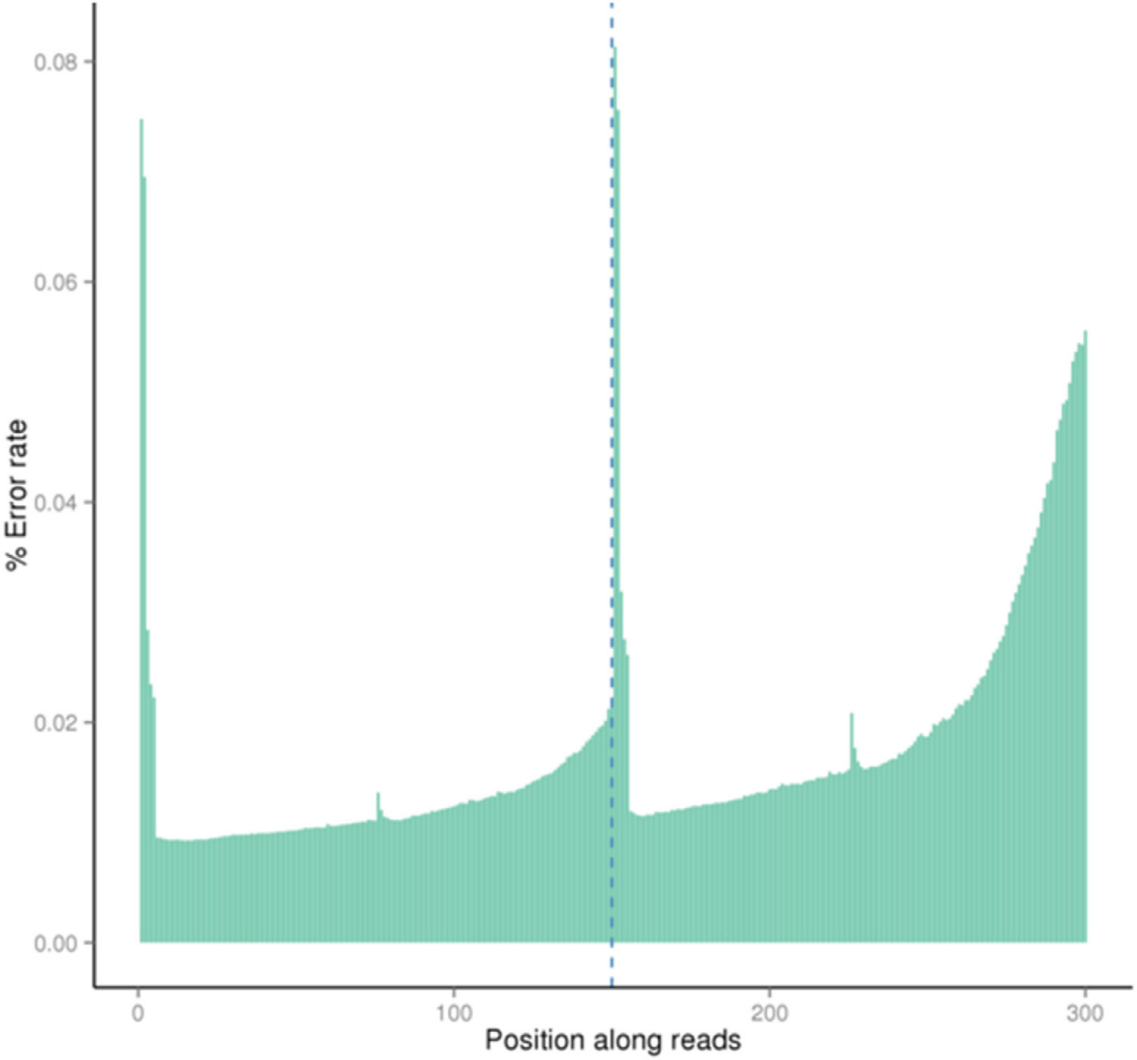
Error rate distribution along reads position.

**Fig. 3:**
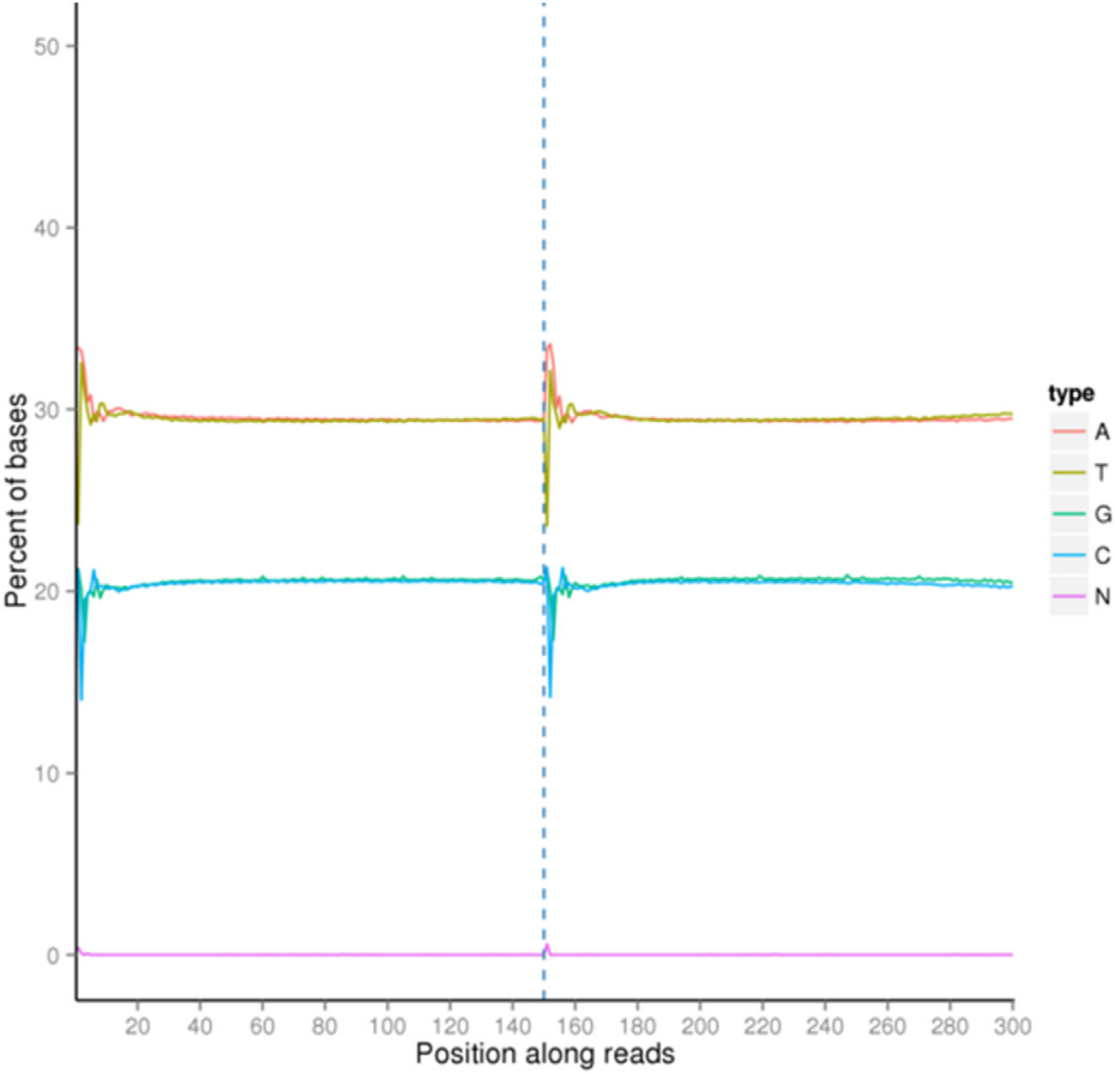
Bases content along reads position.

**Fig. 4:**
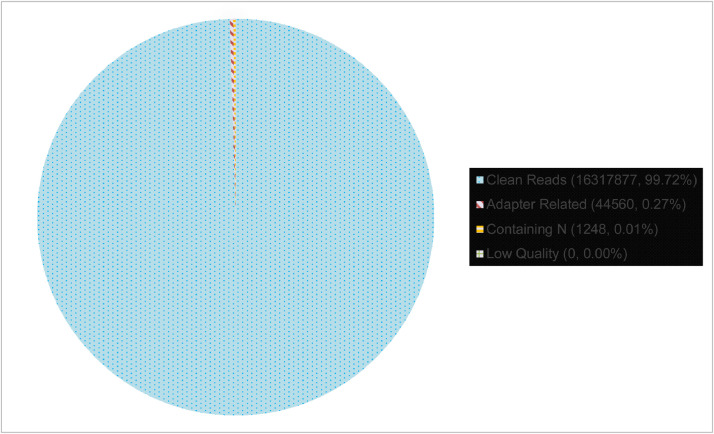
Classification of raw reads.

The quality score of the dataset falls between Q30 to Q40, where Q30 indicates 99.9% of the correct base and Q40 indicates 99.99% of the correct base (Fig. 1). The rate of the single base error along the position of the read is under 0.08% (Fig. 2). The total GC content stands for 40.98% (Fig. 3). Out of 16,363,685 raw reads, 99.72% are the clean reads, followed by 0.27% reads related to the adapter sequence and 0.01% reads containing N base sequence (Fig. 4). The forward adapter sequence is 5′-AATGATACGGCGACCACCGAGATCTACACTCTTTCCCTACACGACGCTCTTCCGATCT-3′ and the reverse adapter sequence is 5′-GATCGGAAGAGCACACGTCTGAACTCCAGTCACATCACGATCTCGTATGCCGTCTTCTGCTTG-3′.

## 2 Experimental design, materials, and methods

### 2.1 Sampling and DNA extraction

The *Tapinoma indicum* was collected using a baiting method with peanut butter and honey [1]. The baits were left for 3 h at the location chosen for baiting. After collection, the *T. indicum* were immediately freeze killed and stored in 95% ethanol under −20 °C. A total of five *T. indicum* workers were used for the genomic DNA (gDNA) extraction. The abdomen body part of *T. indicum* workers were removed before gDNA extraction was performed to minimize the risk of DNA contamination by the gut microbiomes [2]. The gDNA extraction was carried out using HiYield PlusTM Genomic DNA Mini Kit (Blood/Tissue/Cultured Cells) (Real Biotech Corp., Taipei, Taiwan) according to the manufacturer's instruction, with minimum modification by repeating the elution step twice with 50 μl elution buffers to maximize DNA yield. The head and thorax tissues were vortexed in lysis buffer with Proteinase K and incubated at 60 °C for 1 h. After the DNA binds to the filter column through an ethanol wash, elution was carried out twice using 50 μl elution buffers to get a total of 100 μl gDNA solution [3]. The gDNA extracted was quantified by using NanoDrop 2000 c Spectrophotometer (Thermo Fisher Scientific, Massachusetts, US).

### 2.2 Library preparation and sequencing

The sequencing library was generated using NEBNext^Ⓡ^ DNA Library Prep Kit (New England Biolabs, Ipswich, England) following the manufacturer's recommendations. A total of 1.0 µg gDNA was used in DNA fragmentation by randomly shearing into a 350 bp DNA fragment. The DNA fragment was end-repaired and added to dA-tailed. Then the NEBNext adapters for Illumina sequencing were ligated to the DNA fragments and PCR amplified using P5 and indexed P7 oligos. After the purification of the PCR products using the AMPure XP system (Beckman Coulter, Indianapolis, US), the library sequences were analysed for size distribution using Agilent 2100 Bioanalyzer (Agilent, Santa Clara, US) and quantified through real-time PCR. The qualified libraries are pooled and fed into the Illumina Hiseq 2000 sequencers with the layout of pair-ended 150 bp reads.

## Declaration of Competing Interest

The authors declare that they have no known competing financial interests or personal relationships which have, or could be perceived to have, influenced the work reported in this article.

## Appendix Supplementary materials

Image, application 1
